# Applications of bacteriophages versus phage enzymes to combat and cure bacterial infections: an ambitious and also a realistic application?

**DOI:** 10.1007/s00253-018-8811-1

**Published:** 2018-02-13

**Authors:** Barbara Maciejewska, Tomasz Olszak, Zuzanna Drulis-Kawa

**Affiliations:** Department of Pathogen Biology and Immunology, Institute of Genetics and Microbiology, University of Wroclaw, S. Przybyszewskiego 63/77, 51-148, Wroclaw, Poland

**Keywords:** Phage therapy, Phage-borne polysaccharide depolymerases, Phage lysins, Phage enzymes application in treatment

## Abstract

Bacteriophages (phages) are viruses that infect bacteria. The “predator–prey” interactions are recognized as a potentially effective way to treat infections. Phages, as well as phage-derived proteins, especially enzymes, are intensively studied to become future alternative or supportive antibacterials used alone or in combination with standard antibiotic regimens treatment. There are many publications presenting phage therapy aspects, and some papers focused separately on the application of phage*-*derived enzymes. In this review, we discuss advantages and limitations of both agents concerning their specificity, mode of action, structural issues, resistance development, pharmacokinetics, product preparation, and interactions with the immune system. Finally, we describe the current regulations for phage-based product application.

## Introduction

Bacteriophages (bacterial viruses) are obligatory parasites propagating in bacterial hosts. The vast majority of discovered phages belong to dsDNA tailed viruses (*Caudovirales*) and can be distinguished into lytic and temperate phages. Each of these propagation strategies leads to the spread of viral DNA in a different way. Lytic phages are considered as professional host killers, whereas the temperate phages integrate within the host genome, what is often beneficial for the bacterial cell (lysogenic conversion) (Salmond and Fineran 2015). Phages are the most abundant biological particles in the world and playing a significant role in the environment being responsible for (1) dissolved and particulate organic matter circulation via host cell lysis, (2) regulation and biodiversity of populations by reducing the number of dominating bacteria, (3) horizontal gene transfer (HGT) via transduction, or indirectly via transformation of bacterial DNA released during cell lysis, and finally, (4) lysogenic conversion by temperate phages (Wommack and Colwell 2000; Brussaard et al. 2008). Therefore, phages greatly affect microbial diversification as an integral part of each ecological niche including the human body. The tremendous dynamics of the phage–host interactions results in the continuous flow of genetic material, which drives the co-evolution of both entities (Thierauf et al. 2009).

### Phage life cycles—crucial differences

**Fig. 1:**
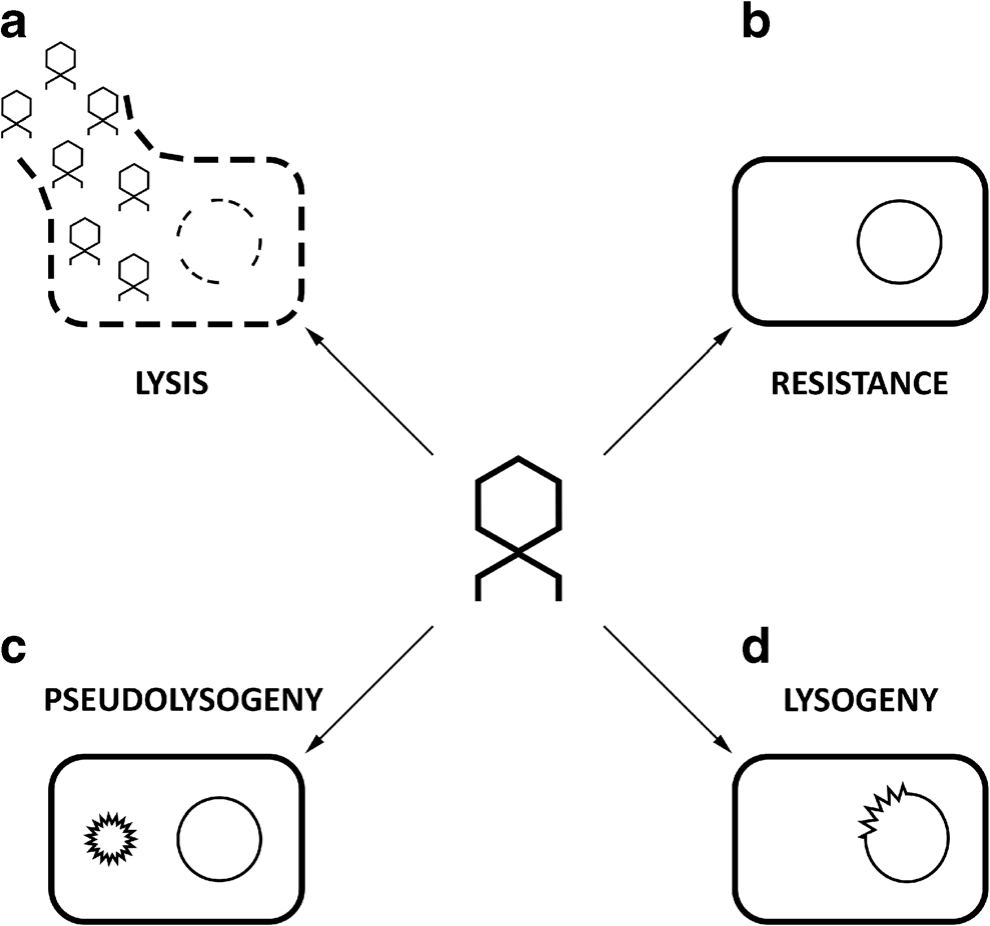
The possible consequences of phage infection: **a** bacterial host lysis and release of phage progeny; **b** lack of virus propagation conditioned by bacterial resistance to phage infection; **c** lack of host lysis and phage DNA maintenance as an episome (pseudolysogeny, lytic and temperate phages); **d** lack of host lysis and phage DNA integration into bacterial genome (lysogeny, temperate phages)

There are three types of life cycles in *Caudovirales*: lytic, lysogenic, and pseudolysogenic (Fig. 1). The typical lytic phage infection consists of six different stages and begins with the adhesion of viral particle to the surface of bacterial cell. Right after adhesion, phage activates various molecular mechanisms leading to the injection of viral DNA into the host cell. The host metabolism is hijacked to amplify viral DNA and to produce phage proteins. Consequently, phage capsids are assembled and packed with genetic material. After the host cell lysis, the phage progeny is released to the environment (Salmond and Fineran 2015).

Temperate phages can propagate in two different ways, either in the lytic strategy or by simultaneous propagation with the cell host as a prophage (lysogeny). The implementation of lysogenic or lytic cycle is governed by several phage-encoded repressors and regulators (e.g., λ phage CI protein), as well as specific phage enzymes such as integrases and excisionases. If environmental conditions stay favorable to the bacterial host, the repressor maintains the phage in lysogenic state. Under stress conditions, bacterial cells may mobilize the SOS response system (especially RecA co-protease) and inactivate the phage repressor, which triggers the expression of lytic cycle genes (Kim and Ryu 2013). A recent report proved the existence of a molecular phage quorum sensing based on the concentration of “arbitrium” molecule, which informs about the current state of phage population accumulation in a particular niche. At the low extracellular concentration of arbitrium, phages propagate intensively in the lytic cycle, whereas increasing number of arbitrium molecules switch lytic cycle to lysogenic (Erez et al. 2017).

The third type of phage existence is pseudolysogeny when the viral DNA is present within a host cell as an independent episome (plasmid-like form). The host is thus only a phage-carrier and the episome segregates asymmetrically during cell division. Formerly, pseudolysogeny was considered as a temporary suspension of phage developmental cycle preventing the release of phage progeny into environment deprived of the sensitive host cells. It should be emphasized that both lytic and temperate phages may undergo pseudolysogeny event and in some cases episomal genes can be expressed influencing host metabolism (Los and Wegrzyn 2012; Krylov et al. 2012; Latino et al. 2016; Argov et al. 2017).

### Phage-based therapy—how did the story begin?

Phages were discovered in 1915 by Frederick William Twort and the term bacteriophages was coined by Felix d’Herelle, who in 1917 independently confirmed Twort’s discovery (Kutter et al. 2010). Phages were immediately recognized as potential antibacterials and used for the treatment of bacterial infections during the 1920s and 1930s. However, phage therapy was abandoned in favor of antibiotics exhibiting a broad activity against bacteria, and being easy to prepare, store, and distribute (Kutter et al. 2010). The benefits of antibiotics and chemotherapeutics were substantially lost in subsequent years following the emergence and dissemination of bacterial drug resistance. Today, multidrug-resistant (MDR) bacterial strains are a serious problem both in hospitals and community settings. Most frequent and especially difficult-to-treat MDR bacteria belong to the so-called “ESKAPE” group and include *Enterococcus faecium*, *Staphylococcus aureus*, *Klebsiella pneumoniae*, *Acinetobacter baumannii*, *Pseudomonas aeruginosa*, and *Enterobacter* spp*.* The pharmaceutical pipeline of antibiotics active against ESKAPE is extremely limited. This group spans methicillin-resistant *S. aureus* (MRSA), vancomycin-resistant enterococci (VRE), as well as carbapenemase (MBL, KPC, OXA-48) and extended spectrum beta-lactamase (ESBL) producers (http://www.eucast.org/resistance_mechanisms/). The emergence of infections caused by MDR pathogens generated a critical need to find alternatives to classical antibiotics (Barrow and Soothill 1997; Alisky et al. 1998; Carlton 1999; Sulakvelidze et al. 2001). For this reason, the phage therapy gets revitalized.

**Fig. 2:**
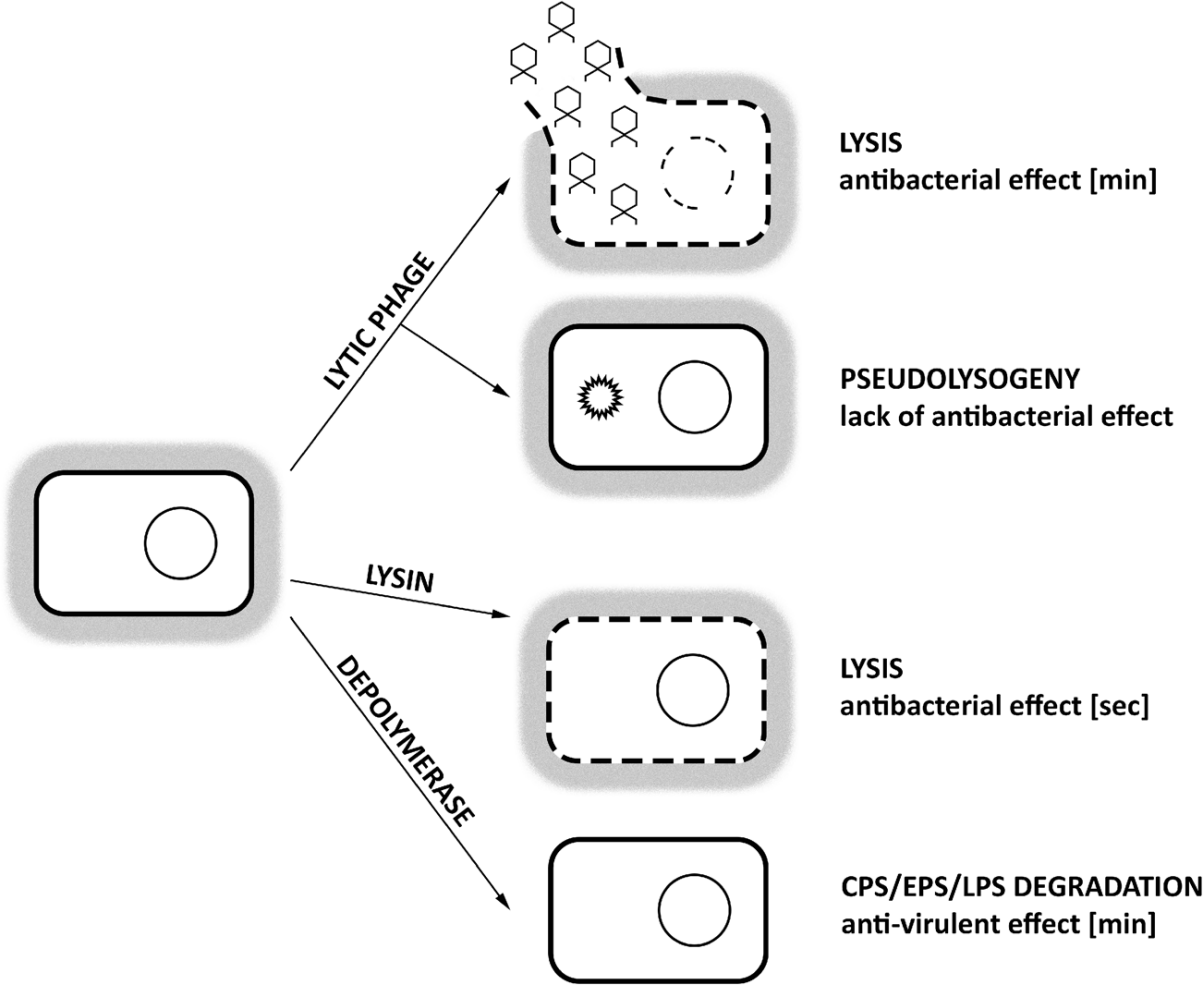
The main characteristics of phage-based products application. The application of lytic phage preparation may result in both bacterial host lysis (the effect observed even in minutes) or lack of lysis (transition into a pseudolysogeny state). The application of lysin-based product leads to the lysis of targeted host (the effect observed even in seconds). The application of depolymerase-based product leads to degradation of capsule (CPS), exopolysaccharides (EPS), or lipopolysaccharides (LPS) decreasing bacterial virulence followed by enhancement of immune system clearance (the effect observed even in minutes)

Considerable interest arose on phage-encoded proteins with antibacterial potential (Fig. 2). These include viral enzymes such as endolysins, virion-associated lysins (VALs), and polysaccharide depolymerases. Endolysins are the lytic enzymes used by phages at the end of the replication cycle to degrade bacterial peptidoglycan (PG) from within, resulting in a rapid host lysis and the release of phage progeny. VALs and depolymerases are linked to the virion particle and serve at the beginning of infection to overcome bacterial cell surface barriers. VALs are responsible for PG degradation required for phage genetic material injection to the infected host cell, whereas depolymerases degrade polysaccharide molecules such as capsule, lipopolysaccharide (LPS), or biofilm matrix (Nelson et al. 2012; Schmelcher et al. 2012a; Rodríguez-Rubio et al. 2013; Latka et al. 2017).

### Phage therapy—issues to consider

#### Specificity and host range

Unlike wide spectrum antibiotics, phage therapy is characterized by selectivity (Table 1). The specificity of phages results from their relatively narrow host range limited usually to one bacterial species. The number of bacterial strains infected by particular phage varies depending on the type of surface receptor recognized and antiviral defense mechanisms by the targeted host. Wide host range phages that propagate on a large number of strains are generally more useful for therapy (Sulakvelidze et al. 2001).

**Table 1:** Major features of lytic bacteriophages and phage enzymes as antimicrobials

Selected features	Phage	Phage enzymes
Specificity and host range	Propagation on bacterial host (predator–prey relation); narrow host range, very specific mostly on one bacterial species	Narrow or broad depending on the chemical composition commonness of targeted macromolecule
Mode of action	Bacteriolytic; phage titer-dependent killing; virulence efficacy: multiplicity of infection (MOI), burst size, propagation rate; effective on growing cells	Bacteriolytic (lysins) or antivirulent (depolymerases); concentration-dependent activity; minimum inhibitory concentration (MIC); effective on growing and non-growing cells
Biofilm eradication	Relatively effective; phage penetration within the biofilm matrix enhanced by virion-associated depolymerases	Biofilm matrix degradation by depolymerases and eradication by lysins
Resistance development	Relatively fast by mutation and selection; receptor modification; passive adaptation; restriction-modification system; CRISPR-Cas; bacteriophage exclusion; superinfection exclusion; abortive infection; pseudolysogeny	Relatively fast change of depolymerase targets (phage receptor modification); low level of induced resistance for lysins
Product modification including genetically modified products	Fast and easy isolation of new phages from environmental source; isolation of naturally evolving phages; engineered phages (genetically modified microorganism not approved for therapy)	In silico development by protein data bases exploration; analysis of annotated phage genomes; engineered proteins (approved for therapy)
Influence on normal flora	Load reduction of targeted strain; regulation of microbiome composition	Load reduction/virulence decrease of targeted strain; regulation of microbiome composition
Impact on immune system	Reticuloendothelial system (RES) clearance and immune cellular defense mechanisms; immunogenic (induction of antibodies production)	Immunogenic (induction of antibodies production)
Safety	Possible endotoxin (LPS) and other toxins release during cell lysis	Possible endotoxin (LPS) and other toxins release during cell lysis
Product preparation (purity, concentration, stability at different temperatures, and pH)	Different stability properties dependent on structural protein composition; limitation in densification and purification; large-scale methods need to be adopted	Relatively stable, especially lysins; recombinant protein expression well developed and large-scale methods adopted
Formulations and delivery route	Liquid phage filtrate, injections, aerosols, tablets, formulas for local application. Parenteral route; orally; locally (topical infections)	Injections, aerosols, formulas for local application; parenteral route; oral application limited by proteolysis; locally (topical infections)
Pharmacokinetics	Not well defined; size and capsid protein composition affects blood and systemic concentration regulated by reticuloendothelial system’s clearance and immune cellular defense mechanisms; self-replicating agent and the concentration increase at the infection site	Well defined for each protein; chemical structure affects penetration, plasma protein binding, and proteolysis degradation—effective concentration; concentration at the infection site related on the systemic concentration and blood circulation
Combined therapy	Cocktail of phages (3–5) or phage–protein; antibiotic–phage–protein combination; prevention of resistance development; extended activity spectrum; synergistic effect possible	Combined therapy of protein–protein; phage–protein; antibiotic–protein; antibiotic–phage–protein; prevention of resistance development; extended activity spectrum; synergistic effect possible

#### Mode of action

One of the basic principles to select phages for therapy is excluding temperate phages because the bactericidal effect is only guaranteed for lytic phages. The most important factor ensuring the effectiveness of the treatment is the self-replicating nature of phages, which distinguishes them from conventional antibiotics. In addition to high burst size and propagation rate, the phage titer and MOI (multiplicity of infection—the number of phage particles per one bacterial cell) are critical factors. Since phages propagate only on actively growing host cells, the high MOI prevents the loss of antibacterial potential associated with phage adhesion to dormant and dead cells or cellular debris (Abedon 2016a).

#### Biofilm eradication

The most common cause of failure of antibiotic therapy in chronic infections is the ability of the bacteria to produce biofilms. Due to the impermeability of biofilm matrix and the clonal diversity of bacterial cells within this structure, the application of standard antibiotics usually fails. The activity of phage preparations against biofilm-forming bacteria is relatively high. Some phages are naturally equipped with virion-associated depolymerases that degrade the biofilm matrix (Lu and Collins 2007; Abedon 2015). Phages can also infect metabolically dormant bacteria if the surface receptor is still present. In this case, the lytic cycle stays suspended until bacteria switch from persistence to active growth (Pearl et al. 2008). However, the mature biofilm is a complex structure and its complete eradication by one phage is rather unlikely. Biofilm elimination takes time and requires the application of multi-phage cocktails or antibiotic supplementation (Abedon 2016b; Chaudhry et al. 2017). The ability of phages to biofilm degradation results from the existence of selective pressure in particular area, where depolymerase degrades matrix exopolysaccharides enabling the phage or other antimicrobials (combined therapy) to reach the bacterial cell (Abedon 2016b).

#### Development of resistance

Bacterial resistance to phage infection was documented by Felix d’Herelle at the very beginning of phage therapy. The interactions between phages and their hosts are commonly described as a parasite–host or predator–prey and both are subjected to the evolutionary mechanisms outlined in the “Red Queen” hypothesis (van Valen 1973). The bacterial resistance to phages can arise in several ways. The most common form is receptor modification due to point mutations of receptor-encoding genes or changes in their expression, which ultimately prevent phage adsorption. Discussing this common phage-resistance mechanism (loss/modification of phage receptor), it must be stressed out that most phages target bacterial surface molecules, especially those of carbohydrate nature. Surface glycans and glycoconjugates such as capsules and LPS serve as molecular patterns for recognition by the innate immune system, and also provide shields to antibiotic entry and host defense mechanisms (e.g., complement system and phagocytosis). Loss or alteration of these molecules could result in bacteria more susceptible to host clearance mechanisms by the immune system. Another resistance mechanism to phage infection is superinfection exclusion systems encoded by other prophages already present in the target bacterial cell, which protect bacteria against infection by other closely related phages. Bacteria may also activate restriction-modification systems, which are responsible for destruction of invading foreign DNA. The more complex mechanism of resistance, operating at the DNA/RNA level, is CRISPR/Cas system, also called the acquired immunity of bacteria (Labrie et al. 2010). Another multi-threaded mechanism protecting bacteria against lytic and temperate phage infection is the bacteriophage exclusion system (BREX), which inhibits foreign DNA replication (Goldfarb et al. 2015). The last resort for resistance mechanism, which operates in the context of the entire population, is the abortive infection system. This mechanism leads to the death of the invaded host cell, preventing phage multiplication and further infection of susceptible population (Labrie et al. 2010). The appearance of phage-resistant bacterial clones cannot be avoided since it is a natural mechanism of bacteria-virus co-evolution, which also occurs in phage therapy. To enhance the abundance reduction of pathogenic strain in treated patient, the polyvalent phage cocktails are composed (Ormälä and Jalasvuori 2013). Nevertheless, recent in vivo studies show that the emergence of phage-resistant mutants does not affect the effectiveness of therapy in immunocompetent patients, where both phage-sensitive and phage-resistant population were cleared out by innate immune mechanisms, especially neutrophils (Bull et al. 2002; Roach et al. 2017).

#### Product modification

Phage preparations used for a specific infection (e.g., wound infection) or against a particular bacterial group (e.g., anti-staphylococci) are usually composed of a multi-phage cocktail, which may be further modified by adding more phages to an existing cocktail or replacing one phage with another. These improvements can be made by selecting phage from an existing collection or by isolating new phage from the environment (Goodridge 2010; Chan and Abedon 2012). Although the molecular engineering tools are currently available to create genetically modified phages, their use is not permitted for human therapy by the Food and Drug Administration or the European Medicines Agency. Nevertheless, there is interest in creating genetically modified temperate phages or lytic phages equipped with specific dedicated biofilm-degrading enzymes, increasing the effectiveness of treatment for chronic, biofilm-related infections (Lu and Collins 2007; Edgar et al. 2012).

#### Influence of phages on normal flora

The narrow host range of phages ensures that phage therapy plays does not adversely affect the natural microbiota. Phage cocktails specific to different bacterial strains or species usually do not contain phages capable of infecting saprophytic bacteria. Nevertheless, the microbiota of each person differs and some people may be the carriers of potentially dangerous species (e.g., multidrug-resistant ESKAPE representatives). In this unique situation, the therapy directed against those potentially pathogenic bacteria may cause imbalance in the microbiota (Loc-Carrillo and Abedon 2011).

#### Impact on immune system

The success of phage therapy largely depends on the patient’s immune system. The interactions between phages and the immune system should be considered in various ways. First, the immune system may recognize and inactivate viral particles (Górski et al. 2012). In vertebrates, the effectiveness of phage clearance depends on the structure of viral capsid (Merril et al. 1996). Even minor changes in phage coat protein composition can affect their bloodstream circulation time and immunogenicity. Fast clearance of phage particles is carried out by the reticuloendothelial system, especially in the liver and spleen. Kupffer cells (macrophages located in the liver) engulf phages four times more efficiently than splenic macrophages. This phenomenon is probably related to the different function of these organs. Kupffer cells are meant to purify the blood of the most serious microorganisms, immune complexes, and cellular debris, whereas splenic macrophages are more involved in stimulating lymphocytes to antibody production (Dabrowska et al. 2005; Górski et al. 2012). Low level of anti-phage antibodies can naturally occur in patients, but their titer against particular phages may increase during phage therapy. Interestingly, the vertebrate immune system does not trigger a specific cellular response against bacteriophages (T cells do not participate in phage elimination) (Górski et al. 2012; Cisek et al. 2017). In addition, the activation of immune responses associated with phage proteins may also exhibit immunomodulating properties. Phages affect phagocytosis and the development of an inflammatory response, but depending on the phage preparation (species, dose, purity, and route of administration) they can either intensify or inhibit these processes (Górski et al. 2012).

#### Safety

The onset of phage therapy dates back to the early twentieth century. Due to the ease of administration and no side effects, phages were used as oral and topical preparations. Despite the primitive methods of purification, the first attempts at intravenous administration of phage preparations began in the mid-1920s (Smith 1924; D’Herelle 1931). The first clinical trial of intravenous therapy was effective despite the occasional adverse effects of a “specific therapeutic shock” (Hugh Young reaction). The elimination of peptone and other animal protein components from the propagation medium reduced the negative effects of phage injections. The introduction of routine phage purification in cesium chloride density gradient, ammonium sulfate precipitation, and filtration on anion-exchange diethylaminoethyl cellulose columns (DEAE) eliminated or significantly diminished potential hazards (Abedon et al. 2011; Speck and Smithyman 2016). Although phage therapy is generally considered safe, its use in immunocompromised patients may be riskier and less effective (Speck and Smithyman 2016; Roach et al. 2017). Another aspect of safety issues is the probability of HGT carried out by phages. Because phages multiply at the site of infection, there is always the risk of some form of HGT that might affect/increase the virulence of co-existing bacterial population or introduction of new antibiotic resistance genes into the population (Lin et al. 2017). The safety of phage therapy in the context of rapid release of bacterial toxins (especially LPS) might be also considered. During phage therapy of Gram-negative bacterial infections, especially using a high dose of phages, a simultaneous lysis of high numbers of bacteria may release endotoxins in such quantities that they might cause endotoxic shock. However, a similar outcome could be also be possible during bactericidal antibiotic (e.g., β-lactams) treatment (van Langevelde et al. 1998).

#### Product preparation

Each step of phage preparation must be strictly controlled to ensure safety. From the very beginning, at the stage of host selection, special attention must be paid to the exclusion of bacterial strains carrying phage-related entities (prophages, satellite phages, episomes containing viral DNA). This will prevent the contamination at the initial stage of production and will reduce the risk of HGT (Abedon 2017). While the multiplication of phages is not complicated, the lysate purification could be troublesome. For the safety issues, the lysate should be purified from toxic products of bacterial metabolism and any cell debris, especially endotoxins (LPS). The multi-stage purification procedure involves centrifugation, filtration (0.22 μm pore filters), organic solvents treatment (chloroform, *n*-butanol, 1-octanol), condensation (polyethylene glycol precipitation, ultracentrifugation in cesium chloride gradient), and dialysis (Bonilla et al. 2016). Due to the wide variety of phage particles, there is no universal protocol for their purification. Phage capsule morphology differences often affect the stability of preparations and the sensitivity of phages to various chemical and physical factors (Alper 1954). Some phage particles are rapidly inactivated by chloroform, others can be damaged during ultracentrifugation or dry freezing processes, and others have a very short shelf-life. On the other hand, there are also phages resistant to high salt concentrations, extreme temperatures or pH values, and long-lasting drying (Jończyk et al. 2011). All above factors make it difficult to obtain a pure phage preparation and to maintain viral particles infective.

#### Formulations and delivery route

As phages are easily propagated in bacteria cultivated in liquid media, these formulations are the most popular form of phage preparations. Moreover, liquid formulations prevent phages from drying and inactivation. Liquids can be administered by the oral, intravenous, or topical route. Phages can be used to prepare aerosols (inhalants), creams/ointments (for topical applications), moist dressings and tampons, and even powders and tablets (Abedon et al. 2011; Weber-Dabrowska et al. 2016). Phage preparations can also be administered intravenously, intramuscularly, vaginally, rectally, or by inhalation. Above methods allow for treatment of many types of infection including gastrointestinal, respiratory, urinary, and even sepsis. Phages easily penetrate from the intestine to the blood and urinary tract, but their delivery to peripheral tissues is usually not sufficient. The transfer of phages through the blood–brain barrier is sometimes problematic. The blood–brain barrier limits passive diffusion between the blood and the brain compartments even for large proteins (> 400 kDa), making it permeable for phages only in the case of blood–brain barrier dysfunction or inflammatory conditions (Weiss et al. 2009). For localized infections such as sinusitis, pharyngitis, or skin infections, the best efficacy is obtained by topical application of aerosols/suspensions or creams/ointments (Letkiewicz et al. 2010; Abedon et al. 2011; Weber-Dabrowska et al. 2016).

#### Pharmacokinetics

The pharmacokinetics of phage preparations depends on many factors. Important aspects are the size of the phages and the structure/composition of their capsids. Phage capsular proteins can interact in various ways with enterocytes and immune cells (especially dendritic cells). The number of phage particles that enter the body fluids depends on the initial phage titer, its resistance to gastrointestinal conditions (pH, digestive enzymes), and the rate of penetration trough the intestinal epithelium. In most of the cases, phages easily cross the barrier of gut epithelium and reach the bloodstream, but their persistence in the circulation varies depending on the efficacy of reticuloendothelial system clearance. In addition, phage particles are also removed by secretions, which in some way facilitates the possible treatment of urinary tract infections. Since the vertebrate immune system may produce Ab specific for phages, this may additionally cause phage inactivation and its elimination. An indirect solution for this problem is the application of phage cocktails composed of relatively distantly related phages, which prevent the cross-reactivity of emerging Ab (Dabrowska et al. 2005; Skurnik and Strauch 2006; Gorski et al. 2006).

#### Combined therapy

At the very beginning of phage therapy age, Felix d’Herelle noted that the effectiveness of single phage therapy rapidly decreased. To maintain a high bactericidal efficacy, it is necessary to use polyvalent phage preparations, composed of phages recognizing several different bacterial receptors. This reduces the risk of therapy failure due to inactivation of phages by the immune system as well as the emergence of phage-resistant strains (Chan et al. 2013). The combination of phages with antibiotics can also have positive effects. Synergism is especially seen in infections caused by biofilm-producing bacteria (Chaudhry et al. 2017).

### Therapy using phage enzymes—issues to consider (Table 1)

#### Specificity and host range

Specificity and host range of PG degrading lysins (endolysins and VALs) vary and depend on protein characteristics as well as on phage species/genus from which the protein is derived (Paul et al. 2011; Rodríguez-Rubio et al. 2013; Latka et al. 2017). Lysins encoded by Gram-positive-specific phages have evolved along its target which is characterized by a strong variation in the peptide composition, crosslinks, and modification of glycan chain (Schleifer and Kandler 1972). Therefore, the activity of those enzymes is limited to certain bacterial species or even serotype (Table 1). This narrow specificity allows for selective killing of a given target pathogen, saving accompanying microflora and reducing the risk of resistance development (Borysowski et al. 2006). In contrast, PG of Gram-negatives has a highly conservative structure with significant similarities shared among different species. Therefore, endolysins and VALs are usually active against a wide host range (Briers et al. 2007; Latka et al. 2017). The third type of antibacterial enzymes (depolymerases) shows high substrate specificity as bacteria can produce a huge diversity of glycans such as capsule (CPS, K-serotype), O-polysaccharide chains (LPS, O-serotype), or extracellular polysaccharides (EPS). Therefore, glycan-degrading phage depolymerases can be useful even for targeting or detecting particular bacterial serotype (Latka et al. 2017).

#### Mode of action

Phage-encoded lysins fall into two major classes according to their mechanism of action: (1) hydrolases degrading PG bonds via hydrolysis and (2) lytic transglycosylases, cleaving glycoside bonds in glycan chain forming 1,6-anhydro ring at the *N*-acetylmuramic acid residue (Höltje et al. 1975). Depending on the type of chemical bond that is hydrolyzed in PG, we distinguish (1) amidases hydrolyzing amide bond, (2) endopeptidases cleaving bonds within peptide chains, and (3) glucosaminidases and lysozymes (muramidases) both hydrolyzing glycoside bonds in the glycan chain (Nelson et al. 2012). The effect of the degrading activity of lysins can be manifested in seconds as osmotic lysis of the targeted cell (Fig. 2). Numerous in vivo trials have been conducted proving lysin’s high effectiveness against Gram-positive pathogens, including *Streptococcus pneumoniae*, MRSA, or *Bacillus anthracis* (Table 2). In contrast, those enzymes applied exogenously have limited effect on Gram-negatives because of the outer membrane layer. To date, only a few endolysins (e.g., SPN9CC, PlyF307, and CfP1gp153) were shown to cross the outer membrane and degrade Gram-negative PG when used as external agents (Lim et al. 2014; Lood et al. 2015; Oliveira et al. 2016). Although phage lysins differ vastly in their lytic activities, ranging from 100 to 10^8^ U/mg, they are still recognized as the strongest PG hydrolyzers. Nanogram amounts of PlyC endolysin derived from streptococcal C1 phage clear a bacterial culture within seconds, being several orders more active than any other described PG hydrolase of non-phage origin (Schmelcher et al. 2012a).

**Table 2:** The application of phage-derived enzymes in animal models

Pathogen	Enzyme	Model	Delivery route	Dose used	Outcome	Reference
Phage lysins						
*Streptococcus pneumoniae*	Pal amidase from pneumococcal phage Dp-1	Mouse model of nasopharyngeal colonization	Topical nasal and pharyngeal administration	Single dose of 1400 U or 700 U	Bacteria eradication	(Loeffler et al. 2001)
	Cpl-1 lysozyme from pneumococcal phage Cp-1	Mouse model of bacteremia and nasopharyngeal colonization	Intravenous injection and topical nasal administration	Single dose of 2000 μg	Bacterial eradication; 80% of animals protected from death	(Loeffler et al. 2003)
		Rat model of pneumococcal endocarditis and bacteremia	Intravenous injection	10 mg/kg, followed by a continuous infusion of 5 mg/kg/h for 6 h or 250 mg/kg, followed by continuous infusion of 250 mg/kg/h for 6 h	Bacteria eradication obtained with a high dose (250 mg/kg)	(Entenza et al. 2005)
		Non-invasive mouse model of nasal mucosa infection	Topical intranasal administration	Two doses of 1000 μg	Bacteria eradication in 90% of animals; 100% prevention of acute otitis media	(McCullers et al. 2007)
		Rat model of meningitis	Intracisternal injection	Single dose of 20 mg/kg for intracisternal injection and 200 mg/kg for intraperitoneal administration	Bacterial cfu reduction of 3 orders (intracisternal injection) and 2 orders (intraperitoneal administration)	(Grandgirard et al. 2008)
		Mouse model of pneumococcal pneumonia and nasopharyngeal colonization	Intraperitoneal injection and topical intranasal administration	Multidose treatment of 1 mg	100% of animals protected from death	(Witzenrath et al. 2009)
		Mouse model of pneumococcal pneumonia and nasopharyngeal colonization	Inhalation of aerosolized Cpl-1	Aerosolized single dose of 25 μL	80% of animals protected from death	(Doehn et al. 2013)
	Pal and Cpl-1	Mouse model of sepsis	Intraperitoneal injection	Single dose of 200 μg; 1100 U of both enzymes	Bacteria eradication	(Jado et al. 2003)
	Cpl-711 chimeric lysozyme	Mouse model of bacteremia	Intraperitoneal injection	Single dose of 25–500 μg	100% of animals protected from death	(Díez-Martínez et al. 2014)
*Streptococcus pyogenes*	PlyC amidase, peptidase from streptococcal phage C1	Mouse model of nasopharyngeal colonization	Topical oral and nasal administration	Single dose of different amount of enzyme (250–1000 U)	Bacteria eradication; 100% prevention against streptococcal colonization	(Nelson et al. 2001)
*Streptococcus pyogenes*	PlyPy peptidase from *S. pyogenes* MGAS5005 prophage	Mouse model of bacteremia	Intraperitoneal injection	Two doses of 1 mg	Bacterial cfu reduction of 2 orders; 50% of animals protected from death	(Lood et al. 2014)
*Streptococcus agalactiae*	PlyGBS peptidase, lysozyme from streptococcal phage NCTC 11261	Mouse model of vaginal infection and oropharynx colonization	Topical intravaginal, oral and intranasal administration	Single dose of 10 U	Bacterial cfu reduction of 3 orders (vaginal infection) and 2 orders (oropharynx colonization)	(Cheng et al. 2005)
	PlyGBS90–1 peptidase, lysozyme (modified PlyGBS)	Mouse model of vaginal infection	Topical intravaginal administration	Single dose of 30 nmol	Bacterial cfu reduction of 4 orders	(Cheng and Fischetti 2007)
*Streptococcus pyogenes* and *Staphylococcus aureus* MRSA *Streptococcus suis*	PlySs2 amidase, peptidase from *S. suis* 89/1591 prophage	Mouse model of bacteremia	Intraperitoneal injection	Single dose of 2 mg	94% and 89% of animals protected from death for *S. pyogenes* and MRSA, respectively	(Gilmer et al. 2013)
		Mouse model of nasal mucosa infection	Topical intranasal administration	Single dose of 0,1 mg	Bacterial cfu reduction of > 4 orders	(Gilmer et al. 2017)
*Staphylococcus aureus* MRSA	MV-L amidase, peptidase from staphylococcal phage phiMR11	Mouse model of nasal infection and bacteremia	Topical intranasal administration and intraperitoneal injection	Single dose of 310–500 U	100% of animals protected from death	(Rashel et al. 2007)
	ClyS chimeric amidase, peptidase	Mouse model of nasal infection and bacteremia	Topical intranasal administration and intraperitoneal injection	Single dose of 960 μg in nasal model; single dose of 2 mg in systemic model	Bacterial cfu reduction of 2 orders (nasal model); 88% of animals protected from death (systemic model)	(Daniel et al. 2010)
		Mouse model of skin infection	Topical skin application	Single dose of 1%, 6%, or 10% (wt/wt)	Bacterial cfu reduction of 3 orders (10% dose)	(Pastagia et al. 2011)
	LysGH15 amidase, peptidase from staphylococcal phage, GH15	Mouse model of bacteremia	Intraperitoneal injection	Single dose of 5–100 μg	Bacteria eradication; 100% of animals protected from death for ≥50 μg dose	(Gu et al. 2011a)
		Mouse model of bacteremia	Intraperitoneal injection	Single dose of 50 μg	Bacteria eradication	(Gu et al. 2011b)
		Mouse model of bacteremia	Intravenous injection	Single dose of 50 μg	Bacterial cfu reduction of 4 orders; 100% of animals protected from death	(Zhang et al. 2016)
	P-27/HP endolysin (unknown mode of action) from staphylococcal phage P-27/HP	Mouse model of bacteremia and healthy mice (safety test)	Intraperitoneal injection (for model of bacteremia) and intramuscular, subcutaneous, intravenous, and intraperitoneal injections for safety test on healthy mice	Single dose of 250 μg	Bacterial cfu reduction of 3 orders	(Gupta and Prasad 2011b)
	P128 chimeric VAL amidase, peptidase	Rat model of nasal infection	Topical intranasal administration	Single dose of 100 μg	Bacterial cfu reduction of ≥ 3 orders	(Paul et al. 2011)
	λSA2-E-Lyso-SH3b chimeric peptidase	Mouse model of mastitis	Intramammary infusion	Single dose of 25 μg	Bacterial cfu reduction of 0.63–0.81 orders	(Schmelcher et al. 2012b)
	Ply187AN-KSH3b chimeric peptidase	Mouse model of endophthalmitis	Intravitreal injection	Single dose of 1 μg/eye	Bacterial cfu reduction of 1 order; significant effects in protecting eyes from endophthalmitis	(Singh et al. 2014)
	80αLyt2 amidase, peptidase from staphylococcal phage phi80α	Mouse model of bacteremia	Intraperitoneal injection	Single dose of 200 μg	100% of animals protected from death	(Schmelcher et al. 2014)
	phi11 amidase, peptidase from staphylococcal phage phi11	Mouse model of bacteremia	Intraperitoneal injection	Single dose of 200 μg	100% of animals protected from death	(Schmelcher et al. 2014)
	LysK amidase, peptidase from staphylococcal phage K	Mouse model of bacteremia	Intraperitoneal injection	Single dose of 200 μg	100% of animals protected from death	(Schmelcher et al. 2014)
	2638A amidase, peptidase from *S. aureus* 2854 prophage	Mouse model of bacteremia	Intraperitoneal injection	Single dose of 200 μg	100% of animals protected from death	(Schmelcher et al. 2014)
	LysWMY amidase, peptidase from staphylococcal phage phiWMY	Mouse model of bacteremia	Intraperitoneal injection	Single dose of 200 μg	100% of animals protected from death	(Schmelcher et al. 2014)
	PlyTW amidase, peptidase from staphylococcal phage Twort	Mouse model of bacteremia	Intraperitoneal injection	Single dose of 200 μg	50% of animals protected from death	(Schmelcher et al. 2014)
	phiSH2 amidase, peptidase from *S. haemolyticus* prophage phiSH2	Mouse model of bacteremia	Intraperitoneal injection	Single dose of 200 μg	50% of animals protected from death	(Schmelcher et al. 2014)
	P68 amidase, peptidase from staphylococcal phage phiP68	Mouse model of bacteremia	Intraperitoneal injection	Single dose of ∼ 120 μg	No protecting effect (low solubility)	(Schmelcher et al. 2014)
	SAL-1 amidase, peptidase from the staphylococcal phage SAP-1	Mouse model of bacteremia	Intravenous injection	Two doses of 12.5–25 mg/kg	Bacteria eradication	(Jun et al. 2013)
		Healthy rats and dogs (safety test)	Intravenous injection	Multiple doses of 25–100 mg/kg	No serious adverse symptoms observed	(Jun et al. 2014)
		Healthy monkeys (safety test)	Intravenous injection	Multiple doses of 1–80 mg/kg	No serious adverse symptoms observed	(Jun et al. 2016)
		Clinical trial on healthy male volunteers (safety test)	Intravenous injection	Single and escalating dose of 0.1–10 mg/kg	No serious adverse symptoms observed	(Jun et al. 2017)
*Bacillus anthracis*	PlyG amidase from *B. anthracis* gamma phage	Mouse model of peritonitis and bacteremia	Intravitreal injection	Single dose of 50–150 U	~ 70% of animals protected from death	(Schuch et al. 2002)
	PlyPH amidase from *B. Anthracis* prophage	Mouse model of peritonitis and bacteremia	Intraperitoneal injection	Single dose of 1.2 mg/ml	40% of animals protected from death	(Yoong et al. 2006)
*Acinetobacter baumannii*	PlyF307 lysozyme from *Acinetobacter* phage RL-2015	Mouse model of bacteremia and mouse in vivo catheter model	Intraperitoneal injection and topical injection directly into the catheter under the skin	Single dose of 1 mg for intraperitoneal injection and two doses of 1 mg for topical application	50% of animals protected from death; catheter biofilm reduction	(Lood et al. 2015)
*Pseudomonas aeruginosa*	Artilysin® (PVP-SE1gp146 lysozyme combining a polycationic nonapeptide)	Model of *Caenorhabditis elegans* gut infection	Oral and topical administration	20 μg/ml per well (~ 10 nematodes per well)	40–63% of animals protected from death (in the presence of 0.5 mM EDTA)	(Briers et al. 2014)
Phage depolymerases						
*Escherichia coli* K1	EndoE endosialidase from coliphage E	Neonatal rat model of bacteremia	Intraperitoneal injection	Single dose of 20 μg	100% of animals protected from death	(Mushtaq et al. 2004)
		Neonatal rat model of bacteremia	Intraperitoneal injection	Single dose of 0.25 μg	80% of animals protected from death	(Mushtaq et al. 2004)
*Salmonella* Typhimurium	P22sTsp endorhamnosidase from *Salmonella* phage P22	Chicken model of gastrointestinal infection	Oral administration	Multiple doses of 30 μg	Bacterial cfu reduction of ~ 1 order	(Waseh et al. 2010)
*Klebsiella pneumoniae*	K64dep capsule depolymerase from *Klebsiella* phage K64-1	Mouse model of bacteremia	Intraperitoneal injection	Multiple doses of 18.75–150 μg	100% of animals protected from death	(Pan et al. 2015)
	depoKP36 capsule depolymerase from *Klebsiella* phage KP36	*Galleria mellonella* infection model	Injection into the last pro-leg	Single dose of 280 μg/ml	40% of animals protected from death	(Majkowska-Skrobek et al. 2016)
*Pseudomonas aeruginosa*	LKA1gp49 LPS lyase from *Pseudomonas* phage LKA1	*Galleria mellonella* infection model	Injection into the last pro-leg	Single dose of 0.05–0.5 μg	20% of animals protected from death	(Olszak et al. 2017)

Phage depolymerases are responsible for degrading carbohydrate macromolecules in the bacterial cell envelope. Depolymerases are divided according to their mode of action into (1) hydrolases and (2) lyases cleaving a glycosidic bond by *trans*-β-elimination. The hydrolases comprise sialidases (hydrolyzing internal α-2,8-linkages in capsular polysialic acid), rhamnosidases (cleaving α-1,3 *O*-glycosidic bonds between l-rhamnose and d-galactose in the O-antigen of *Salmonella* LPS), levanases (hydrolyzing β-2,6-bonds between fructose monomers in levan), xylanases (cutting β-1,4 bonds within xylan), dextranases (cleaving α-1,6-linkages between glucose units in dextran), and LPS deacetylases which deacetylate the O-antigen rather than breaking the polysaccharide chain (Prokhorov et al. 2017; Latka et al. 2017). The lyases include hyaluronate lyase (cleaving β-1,4 bonds in hyaluronic acid), pectate lyase (cleaving α-1,4 bonds of polygalacturonic acid), alginate lyase (cutting α-1,4 bonds of alginate), and K5 lyase (cleaving α-1,4 bonds of *E. coli* K5 capsules). Depolymerases as antimicrobials can be successfully implemented as external agents to degrade bacterial capsules, LPS, and exopolysaccharides, acting indeed as anti-virulent agents and sensitizing bacteria to antimicrobials, the immune system, and desiccation (Pires et al. 2016; Latka et al. 2017). Like for endolysins, the therapeutic efficacy of recombinant depolymerases was confirmed in animal models (Table 2).

#### Biofilm eradication

Phage depolymerases have evolved as a response against the thick polysaccharide layer covering bacterial cell and hiding phage receptor required for successful attachment to the host. One of such layer is biofilm matrix composed mostly of exopolysaccharides. Moreover, LPS-degrading enzymes are also able to loosen biofilm structure as LPS-containing outer membrane vesicles are embedded within the matrix (Olszak et al. 2017). There are many reports confirming the efficacy of depolymerases in eradication of biofilms formed by both Gram-positive and Gram-negative bacteria (Mushtaq et al. 2004, 2005; Cornelissen et al. 2011; Gutiérrez et al. 2012, 2015); Bansal et al. 2014; Pan et al. 2015). Besides depolymerases, also phage lysins have been successfully used in the removal of bacterial biofilms. Most of the studies were dedicated to *S. aureus* (Sass and Bierbaum 2007; Son et al. 2010; Fenton et al. 2013; Schmelcher et al. 2014; Singh et al. 2014; Gutiérrez et al. 2014; Yang et al. 2015) and streptococcal biofilm treatment (Domenech et al. 2011; Meng et al. 2011; Shen et al. 2013; Rico-Lastres et al. 2015). Concerning the biofilms of Gram-negative bacteria, endolysins Lys68 (Oliveira et al. 2014), LysPA26 (Guo et al. 2017) and PlyF307 (Lood et al. 2015) were proved to be effective against *Salmonella* Typhimurium, *P. aeruginosa*, and *A. baumannii*, respectively.

#### Development of resistance

Phage lysins are in a certain sense unique relative to whole phages and antibiotics since resistance is an extremely rare event. This is due to lysin’s ability to bind and cleave highly conserved targets within the cell wall (Fischetti 2010). Moreover, high specificity of most endolysins reduces the probability of developing bacterial resistance (Fischetti 2005). Nevertheless, secondary modifications of bacterial cell walls, such as *O*-acetylation and *N*-deacetylation in PG or d-alanylation in teichoic acids, can be considered as potential resistance mechanisms against phage lysins, in analogy to what was reported for human lysozyme (Vollmer et al. 2008; Guariglia-Oropeza and Helmann 2011). There are some studies addressing the repeated exposure to low concentrations of the enzyme, which proved no resistance phenotypes to native or engineered phage lysins (Loeffler et al. 2001; Schuch et al. 2002; Fischetti 2005; Pastagia et al. 2011; Schmelcher et al. 2012a; Gilmer et al. 2013). Resistance against phage depolymerases develops quite often due to modifications or variations in polysaccharide composition of capsule, exopolysaccharides, or LPS. The application of depolymerase resulted in the rapid emergence of *E. coli* O9:K30 and *Klebsiella* O1:K20 resistant mutants (McCallum et al. 1989).

#### Product modification

Current synthetic biology techniques can be used to improve the efficacy of phage lysins. Random mutagenesis within enzymatic domain (EAD) or the exchange of cell wall binding domain (CBD) can increase lytic activity. The spectrum of enzymes was experimentally extended by (1) the fusion of two full-length endolysins, (2) the addition of a heterologous EAD to a full-length enzyme, (3) the addition of a heterologous CBD to a truncated endolysin, (4) the duplication of CBD, or (5) the combination of two heterologous CBDs (Cheng and Fischetti 2007; Schmelcher et al. 2012a). Recent studies propose the application of genetically modified endolysins (Artilysins®) or enzymes combined with membrane permeabilizers to efficiently destroy PG in Gram-negatives (Briers et al. 2011, 2014; Oliveira et al. 2014; Yang et al. 2015). These modifications involve the attachment of short (6–100 aa) membrane-penetrating or membrane-destabilizing peptides usually of polycationic, hydrophobic, or amphipathic nature causing membrane disruption or pore formation (Briers et al. 2014; Peng et al. 2017). Numerous endolysins (Ply511, PlyA, CD27L, OBPgpLYS) were recently modified this way and patented as Artilysins® (Briers et al. 2014; Schirmeier et al. 2017). In addition to structure-based modification, outer membrane destabilizing agents such as EDTA, weak organic acids (citric acid), and polycationic agents could be mixed with lysin preparation to enhance antibacterial activity against Gram-negatives (Oliveira et al. 2014). There is not much data on the modification of phage depolymerases. One of the main reasons for this is the relatively big size of these enzymes (usually ~ 1000 aa) forming a complicated spatial structure of trimers or sometimes tetramers. Depolymerases being an integral part of phage particle are still not well-studied proteins concerning enzymatic activity and specificity (Latka et al. 2017).

#### Influence on normal flora

A high specificity of lysins targeted to Gram-positives allows for the selective killing of given pathogens with little to no effect on normal human microbiota. Nevertheless, in some cases, phage enzymes may show a broad spectrum as recently reported for enterococcal phage lysin active against enterococci, *S. aureus*, *Streptococcus pyogenes*, and *S. agalactiae* (Yoong et al. 2004). Another example is streptococcal lysin PlySs2, able to eradicate staphylococci, several species of *Streptococcus* (*S. agalactiae*, *S. pyogenes*, and *S. pneumoniae*), and *Listeria* sp. as well (Gilmer et al. 2013). Lysins derived from Gram*-*negatives infecting phages show theoretically a broad spectrum when combined with permeabilizing agents. In this regard, such preparation might influence the accompanying microflora with the same efficiency as for the targeted pathogen.

#### Effect on the immune system

Due to their proteinaceous nature, phage enzymes stimulate a rapid immune response and generation of neutralizing antibodies (Fischetti 2010). Antibodies against Cpl-1, Pal, MV-L, ClyS, and SAL-1 endolysins were confirmed in several animal studies (Table 2) (Jado et al. 2003; Loeffler et al. 2003; Rashel et al. 2007; Daniel et al. 2010; Jun et al. 2014). The first clinical trial on SAL200 preparation (endolysin SAL-1) also revealed anti-endolysin antibodies in collected serum samples (Jun et al. 2017). Although antibodies were poorly effective in lysin inactivation, their presence sufficiently reduced the systemic half-life of enzymes to approximately 20 min (Loeffler et al. 2003). In vitro and in vivo studies on different endolysins and pathogens confirmed that antibodies slow down the antimicrobial efficacy of lysins but do not abolish their activity completely (Loeffler et al. 2003; Fischetti 2005; Rashel et al. 2007; Jun et al. 2014). The modification of lysins to extend their half-life is possible. Attempted dimerization of Cpl-1 endolysin through the introduction of C-terminal cysteine residues and subsequent formation of disulfide bonds resulted in a twofold increase of anti-pneumococcal activity and tenfold reduction of plasma clearance (Resch et al. 2011). Interestingly, a recently described chimeric endolysin ClyS turned out to be completely insensitive to generated antibodies (Daniel et al. 2010; Pastagia et al. 2011).

#### Safety

Phages are an integral part of the natural human microbiota and the constant release of lysins and depolymerases has no adverse effects on human health (Navarro and Muniesa 2017). For this reason, phage enzymes are considered to have a good safety profile, which was confirmed in many trials using animal models (Table 2). The clinical trials on intravenous administration of SAL200, conducted accordingly to good laboratory practice, demonstrated good tolerance in healthy male volunteers (Jun et al. 2017). Phage enzymes, like other proteins, can theoretically induce an allergic response or some adverse side effects, but these have not been reported in animal models (Jado et al. 2003; Gu et al. 2011a; Gupta and Prasad 2011a; Jun et al. 2014; Pan et al. 2015). Both lysins and depolymerases are specific for unique and highly conserved bacterial structures (polysaccharides or PG) that are absent in mammalian cells, and therefore are non-toxic agents. The good safety profile also includes relatively fast biodegradability (Nelson et al. 2012). The side effects of lysin applications are similar to those of lytic phages and bactericidal drugs, and are associated to the release of endotoxin, as well as bacterial cellular contents and debris during rapid cell lysis, especially in the case of massive infections. This release may induce strong immune responses leading to endothelial and tissue damage, and severe hemodynamic and metabolic derangements, namely, toxic shock (Prins et al. 1994; Nau and Eiffert 2002; Ramachandran 2014). The in vivo administration of phage depolymerases (endosialidase, endorhamnosidase, lyase) against *E. coli*, *S.* Typhimurium, or *K. pneumoniae* was effective in killing bacteria and safe (Table 2). There is a lack of controlled clinical trials dealing with the systemic application of phage-based enzymes in the treatment of infected patients. Detailed evaluation including clinical trials of multiple increasing doses and assessing the effects of therapy on vital functions, such as in the respiratory, central nervous, and cardiovascular systems, are necessary to improve our understanding of the safety profile of phage enzymes.

#### Product preparation

Preparation of lysin/depolymerase formulations should not pose any major problems. Methodologies and strategies for recombinant protein production and purification are systematically improved, and well-established conditions allow for rapid preparation of ultrapure protein in a large scale (Wingfield 2015)*.* The phage-based products characterized so far possess the desired formulation parameters; they remain stable at fridge storage temperature (4 °C) for weeks or even months, and can be kept frozen or lyophilized (Cheng and Fischetti 2007; Pastagia et al. 2011; Gilmer et al. 2013; Jun et al. 2013). Protein stability can be further increased by the selection of optimal conditions (protein concentration, buffer, pH, temperature, additional stabilizers) (Jun et al. 2013) or by molecular engineering (Heselpoth et al. 2015). The vast majority of currently described phage-enzymes was relatively stable at wide pH range (Yoong et al. 2006; Maciejewska et al. 2017; Olszak et al. 2017), suggesting that they might remain functional even after oral administration. Several phage-based products showed to be highly thermostable (above 80 °C), a property that extends their application to industry as for instance food preservatives (Matsushita and Yanase 2008; Plotka et al. 2014; Oliveira et al. 2014; Rodríguez-Rubio et al. 2016; Majkowska-Skrobek et al. 2016; Maciejewska et al. 2017; Olszak et al. 2017).

#### Formulations and delivery route

Enzyme-based formulations applied to date are prepared as injections, aerosols for inhalations, and formulas for local application (liquids, ointments, and gels) (Table 2). Numerous commercially available formulations (emollients ointments, petrolatum for topical application, surfactants, or injection buffers like Dulbecco’s phosphate-buffered saline) were applied for phage-based products preparation (Pastagia et al. 2011; Jun et al. 2013). Like phages, the enzyme-based products must be preceded by thorough and multistep protein purification with the removal of bacterial endotoxins. The reduction of endotoxins to a maximum level of 5 U/kg of body weight per hour for intravenous applications is a challenging procedure but crucial for safe therapy (Pan et al. 2015). The aerosolized and topical enzyme delivery ensures a drug direct accumulation at the site of infection with relatively low systemic exposure (Ryan et al. 2011). To date, the in vivo tests covered the following delivery routes of phage enzymes: injections (intravenous, intraperitoneal, and intravitreal), trans-nasal, and vaginal administration, oral delivery, inhalations, topical application, and injection directly under the skin (Table 2). Each of the listed routes provided effective treatment. The enteral delivery of phage proteins poses a challenge to maintain enzyme activity at low pH and in the presence of proteolytic enzymes in the stomach. There is one example of successful oral application of P22sTsp depolymerase insusceptible to trypsin and partially to chymotrypsin activity (Waseh et al. 2010). To avoid this obstacle, phage enzymes could be encapsulated in polymeric nanoparticles and thus protected from the harsh gastric environment (Chan et al. 2010). An inventive strategy proposed to preserve lysins in the gastrointestinal tract involved the administration of engineered lactic acid bacteria excreting the endolysin while colonizing intestines (Mayer et al. 2008; Gervasi et al. 2014).

#### Pharmacokinetics

A successful treatment depends on well-characterized pharmacokinetic/pharmacodynamic properties of the individual medical product. Despite a wealth of knowledge about antibacterial potential and biochemical parameters of phage enzymes, little is known about their capacity to penetrate mammalian tissues and cells, which influences their effective concentration and dose, timing of administration, or optimal treatment duration. Currently, we can only rely on assumptions and scarce reports in animal models (Table 2). Due to the much smaller size comparing to phages itself, phage enzymes should penetrate more efficiently to human tissues. Indeed, the PlyC endolysin can cross the epithelial cell membrane to reach and lyse intracellular *S. pyogenes* (Shen et al. 2016). The effective intravenous, intraperitoneal, or oral administration in animal bacteremia indicated rapid distribution of lysins and depolymerases within the body and a good penetration to adjoining tissues (Table 2). In the clinical trial of SAL200 preparation, the doses provided a maximum concentration of 10 mg/kg of body weight (Jun et al. 2017). The majority of reports described the effectiveness of a single dose of recombinant phage enzyme for infection eradication (Nelson et al. 2001; Jado et al. 2003; Cheng et al. 2005; Mushtaq et al. 2005; Grandgirard et al. 2008; Daniel et al. 2010; Gu et al. 2011a; Doehn et al. 2013; Lood et al. 2015; Majkowska-Skrobek et al. 2016). Nevertheless, the multiple lysin doses increased the systematic drug concentration followed by a significant improvement in animal survival rate (Oechslin et al. 2013; Jun et al. 2016).

#### Combined therapy

Antimicrobial synergy was demonstrated for several lysins and depolymerases in combination with other PG hydrolases, as well as with numerous classes of antimicrobials (antimicrobial peptides, antibiotics). The in vivo synergy of glycopeptides and β-lactams with MV-L (Rashel et al. 2007) and ClyS (Daniel et al. 2010) endolysins was confirmed in the treatment of systemic MRSA infections. Chimeric endolysin λSA2-E-LysK-SH3b acts synergistically with lysostaphin in a mice model (Schmelcher et al. 2012b), similar to the combination of Cpl-1 with Pal endolysins (Jado et al. 2003).

## Summary: applications of bacteriophages versus phage enzymes to combat and cure bacterial infections: an ambitious and also a realistic application?

The rapid development of phage therapy that took place in the 1920s and 1930s significantly slowed down by the invention of antibiotics. Discovery of penicillin led to almost complete abandonment of phage therapy in the Western countries. However, several research centers (in Georgia, Russia, and Poland) continued research on bacteriophages and their cumulative experience is crucial in the present era of rapid antibiotic resistance development. Currently, the use of phages and phage-borne enzymes in the EU and USA is considered as experimental therapy, which can only be applied under the umbrella of the Article 37 of Helsinki Declaration (World Medical Association 2013; Debarbieux et al. 2016).

“In the treatment of an individual patient, where proven interventions do not exist or other known interventions have been ineffective, the physician, after seeking expert advice, with informed consent from the patient or a legally authorised representative, may use an unproven intervention if in the physician’s judgement it offers hope of saving life, re-establishing health or alleviating suffering. This intervention should subsequently be made the object of research, designed to evaluate its safety and efficacy. In all cases, new information must be recorded and, where appropriate, made publicly available.”

There are two different approaches to phage therapy. One focuses on the “*sur-mesure*” therapy individually matching of phages to the bacterial strain isolated from a particular patient. The second one is called the “*prêt-à-porter*” model, which is based on the application of already-made, polyvalent phage cocktail dedicated to the treatment of a particular type of infection or targeted to the selected pathogen. It is difficult to decide which model is better, but legal constraints make the “*prêt-à-porter*” model a little easier to implement today.

In the USA and EU, the phages and phage-based products (enzymes) classified as human therapeutic products are subjected to the same implementation rigors as conventional drugs. That regulation raises some controversy because of the biological nature of phage preparations (especially based on infective phages). The Food and Drug Administration in the USA and the European Medicines Agency do not allow any modifications to finished medicinal products. Thus, the potentially registered phage preparations cannot be improved in any way after approval. In practice, a long and extremely expensive registration procedure results in a product with a very restrictive scope of activity and suitable only for a “*prêt-à-porter*” model. Further, because phage products are classified as Biological Medicinal Products (BMPs), their use is not allowed under the “hospital exemption,” as in the case of Advanced Therapy Medicinal Products (ATMPs). This regulation limits the use of targeted phage therapy designed for a particular patient. In conclusion, the legislative gaps listed above make the large pharmaceutical companies uninterested in developing phage preparations (Verbeken et al. 2016). Despite many institutional and legislative shortcomings, phage therapy is successfully used in EU in the Ludwik Hirszfeld Institute of Immunology and Experimental Therapy of Wroclaw, Poland (Miedzybrodzki et al. 2012), as well as in Queen Astrid Military Hospital in Brussels, Belgium (Jennes et al. 2017). Nonetheless, European law must undergo serious modifications to the status of phage therapy and to the registration of phages and phage derivatives. Otherwise, in the age of an increasing drug resistance, it may not be possible to draw from the advantages of phages as an effective alternative to antimicrobial therapy (Pirnay et al. 2011).

Concerning the application of phage-based enzymes, the preliminary studies involving animal models and clinical trials are demonstrating promising antibacterial efficacy and confirming their safety (Table 2). However, the current regulations also hamper the use of recombinantly produced phage proteins for therapeutic purposes, especially for long-term systemic treatment (Chan and Abedon 2012; Schmelcher et al. 2012a). The main reason is the limited data of phage enzyme interactions with the human body, which will require to perform further detailed studies concerning pharmacokinetic/pharmacodynamic properties. Nevertheless, the first phage-lysin-based preparations for topical applications, i.e., Staphefekt™ (developed by Micreos), is already registered and commercially available (Totté et al. 2017). Moreover, the first clinical trial on anti-staphylococci endolysin (SAL200 preparation) has also started (Jun et al. 2017).
